# Preliminary analyses of scRNA sequencing and immunohistochemistry of children's lung tissues indicate the expression of SARS‐CoV‐2 entry‐related genes may not be the key reason for the milder syndromes of COVID‐19 in children

**DOI:** 10.1002/ctm2.300

**Published:** 2021-05-21

**Authors:** Yue Tao, Ruwen Yang, Chen Wen, Jue Fan, Jing Ma, Qiao He, Zhiguang Zhao, Xinyu Song, Hao Chen, Guocheng Shi, Minzhi Yin, Nan Fang, Hao Zhang, Huiwen Chen, Xi Mo

**Affiliations:** ^1^ The Laboratory of Pediatric Infectious Diseases, Pediatric Translational Medicine Institute Shanghai Children's Medical Center, School of Medicine, Shanghai Jiao Tong University Shanghai China; ^2^ Department of Cardiothoracic Surgery Shanghai Children's Medical Center, School of Medicine, Shanghai Jiao Tong University Shanghai China; ^3^ Singleron Biotechnologies Nanjing China; ^4^ Department of Pathology Shanghai Children's Medical Center, School of Medicine, Shanghai Jiao Tong University Shanghai China; ^5^ Wenzhou Medical University Second Affiliated Hospital Wenzhou China; ^6^ Department of Cardiothoracic Surgery First Affiliated Hospital of Soochow University Suzhou China

Dear Editor,

Accumulating data have suggested that the SARS‐CoV‐2 infection in children is relatively rare and less critical when compared to adults.^1^ However, a recent study demonstrated that children and adults have comparable chances of infection by SARS‐CoV‐2 when exposed to similar environment.^2^ At the same time, the low incidence of critical illness in children has been repeatedly reported and widely accepted despite the underlying mechanisms remaining elusive.^1^ Two major hypotheses regarding the reasons behind the milder symptoms in children during infection were proposed: (1) a relatively lower expression of genes associated with viral‐entry compared to adults and (2) immune‐related factors.^3^ Several lines of evidence have proposed that the immune system of children is still under development and constantly challenged by pathogens, which could lead to trained immunity with an enhanced innate immune function. These factors might thus help explaining the milder symptoms of SARS‐CoV‐2 infected children.^4^, ^5^, ^6^ However, whether the expression levels of viral‐entry genes might contribute to the milder symptoms observed in children has not yet been confirmed. Therefore, in the present study, we analyzed the expression levels of viral‐entry genes (i.e., *ACE2*, *TMPRSS2*, and *FURIN*) in both children and adult lung tissues by single‐cell RNA sequencing (scRNA‐seq) and immunohistochemistry (IHC).

Angiotensin converting enzyme 2 (ACE2) is the key receptor of SARS‐CoV‐2 to gain entry in the cell via its interaction with the spike (S) protein of the virus, which is cleaved by the transmembrane protease serine 2 (TMPRSS2). Importantly, it should be noted that simultaneously blocking the activity of TMPRSS2 and CATHEPSIN B/L cannot completely inhibit the entry of SARS‐CoV‐2 in vitro, suggesting a possible involvement of additional proteases in the priming of SARS‐CoV‐2.^7^ A FURIN cleavage site has been identified at the S1/S2 boundary in the SARS‐CoV‐2 S protein, which has been suggested to be potentially cleaved by FURIN as an additional possible mechanism for the priming of the virus.

In order to determine the expression levels of these three genes, we collected non‐affected lung tissues from 10 children with congenital heart disease combined with lung diseases requiring lobectomies (Figure 1A, Table S1) and analyzed the transcriptome of 125,955 cells via scRNA‐seq. The cells were clustered and annotated into 15 cell types, and we were able to identify major cell types known to exist in the lung (Figure 1B). We observed that the same cell subtypes were present in children and a public dataset (GSE122960) comprised of eight adult lung donors (totally 42,843 cells; Figure 1B). Consistent with previous analyses, AT2 cells (and proliferating AT2 cells) were the major cell type expressing *ACE2* (Figure 1D). Other lung/bronchiolar epithelial cells, including club cells in adults and AT1 cells in children, also showed a significant expression of *ACE2*. Similarly, the AT2, AT1, and club cells were the major cell types with relatively high expression of the *TMPRSS2* gene in both adults and children (Figure 1D). However, the expression patterns of *FURIN* slightly differed from those of *ACE2* and *TMPRSS2*, with vascular endothelial cells and monocytes being the major expressing cell types in adults, and AT1 and AT2 cells in children (Figure 1D). A more detailed analysis focusing specifically on AT2 cells revealed that the average expression levels of *ACE2*, *TMRPSS2*, and *FURIN* were not significantly different between children and adults (*p* = 0.47, 0.95, and 0.11, respectively; Figure 1C, left panel). In terms of the percentage of positive cells in the AT2 population, no significant changes were observed in *ACE2* expressing cells between children and adults (*p* = 0.45. Figure 1C, middle panel). The numbers of *TMPRSS2* and *FURIN* expressing AT2 cells were significantly elevated in adult lungs compared to that of children (*p* = 0.0041 and 0.00062, respectively; Figure 1C, middle panel). However, given the comparable number of cells expressing *ACE2*, such elevation may not lead to more entry of the virus.

**FIGURE 1:**
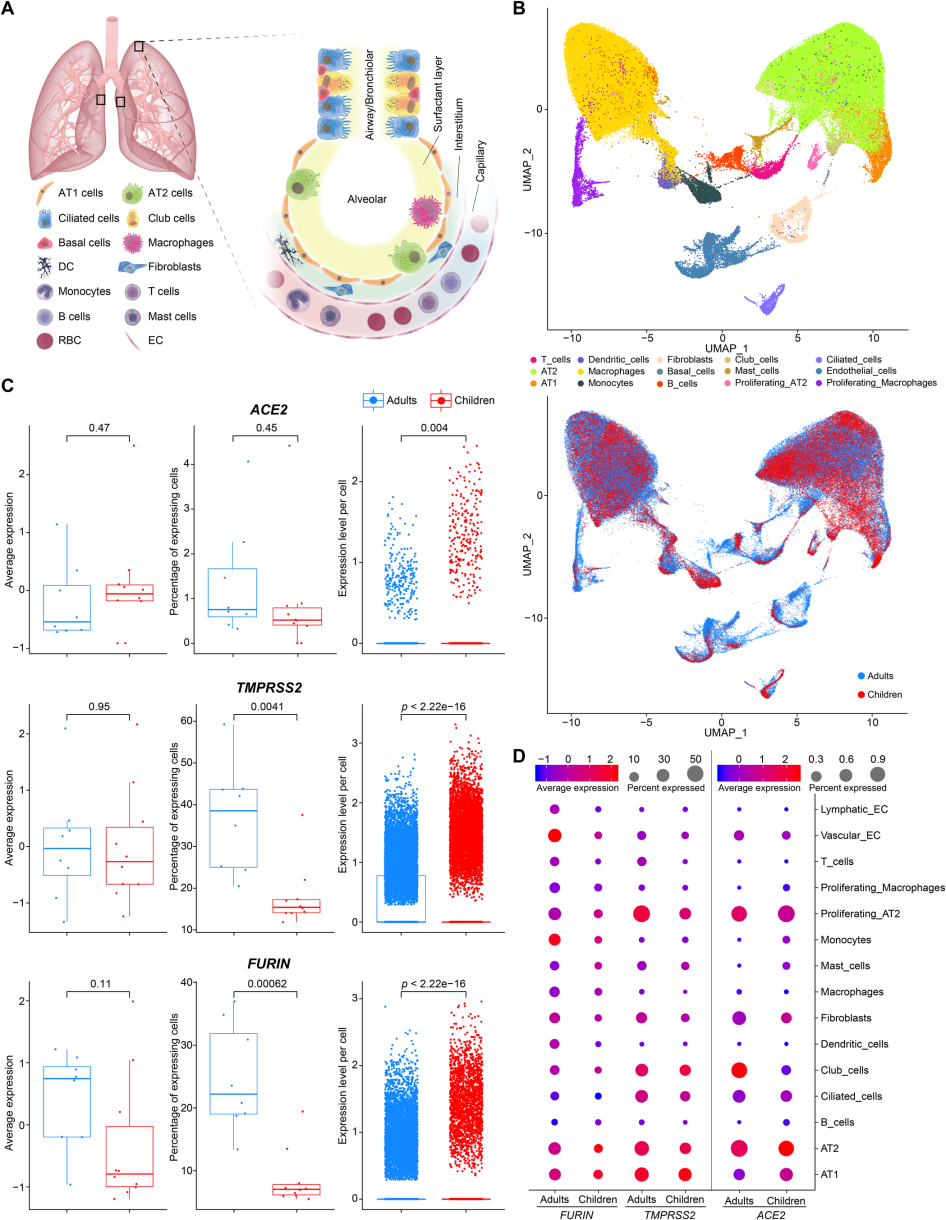
Expression of *ACE2*, *TMPRSS2*, and *FURIN* across different cell types in the lung samples from children and adults determined by scRNA‐seq analysis. (A) Schematic illustration of the representative sampling locations of the surgical lung specimens from children used in this study, and an overview of the major cell types detected in these specimens. The graphic components were obtained and adapted from the Library of Science and Medical Illustrations (http://www.somersault1824.com). (B) UMAP (uniform manifold approximation and projection) visualization of an integrated scRNA‐seq dataset including data from GSE122960, and the sequencing results from 10 children lung specimens. Fifteen clustered and annotated cell types are color‐coded (upper panel), while cells from adults and children are distinguished with blue and red dots, respectively (lower panel). (C) Expression levels of *ACE2*, *TMPRSS2*, and *FURIN* in the AT2 cells of lung samples. Average expression levels in each sample, proportion of expressing cells in each sample, and expression levels of each cell are shown as Box and Whisker Plots (from left to right). A *t*‐test was used for statistical analysis. The level of significance is reflected by the *p* value shown in the plots. (D) Dot‐plot illustrating expression of *ACE2*, *TMPRSS2*, and *FURIN* across all cell types in the lung samples. Dot color represents the Z‐score normalized average gene expression within each particular cell type. Dot size represents the percentage of cells expressing the respective genes within each cell type. Given the extremely low number of basal cells detected in the lung samples of children, these data points were excluded from the dot‐plot to allow for proper visualization of the general view of the gene expression in the majority of the cell types. The complete dataset used for generation of this figure is listed in Table S3. Abbreviations: AT1, alveolar type I; AT2, alveolar type II; DC, dendritic cell; EC, endothelial cell; RBC, red blood cell; SMC, vascular smooth muscle cell.

In order to further verify the expression of *ACE2*, *TMPRSS2*, and *FURIN* at the protein level, IHC was performed in lung biopsy specimens from children and adults in two independent cohorts (Table S2). In a pattern that was consistent with the scRNA‐seq analysis, the overall expression levels of ACE2 in children and adults were comparable in both cohorts, with a very small portion of the lung cells expressing ACE2. However, both TMPRSS2 and FURIN showed higher expression levels in children compared to adults, although the former did not reach statistical significance possibly due to the small sample sizes available (Figures 2A and 2B). The underlying reasons for the partial discrepancy between the results from scRNA‐seq and IHC might be related to the complex processes occurring downstream of transcription, in particular post‐transcriptional, translational, and degradation regulation. Meanwhile, the semi‐quantitative characteristics of IHC and its limitation in identifying cell types may also contribute to the observed discrepancies. This further emphasizes the necessity to combine these methods in order to achieve an impartial interpretation of the results. Moreover, in accordance with a previous report, the staining patterns of ACE2, TMPRSS2, and FURIN were consistent in the same types of tissue regardless of the pathological condition of the patient.^8^

**FIGURE 2:**
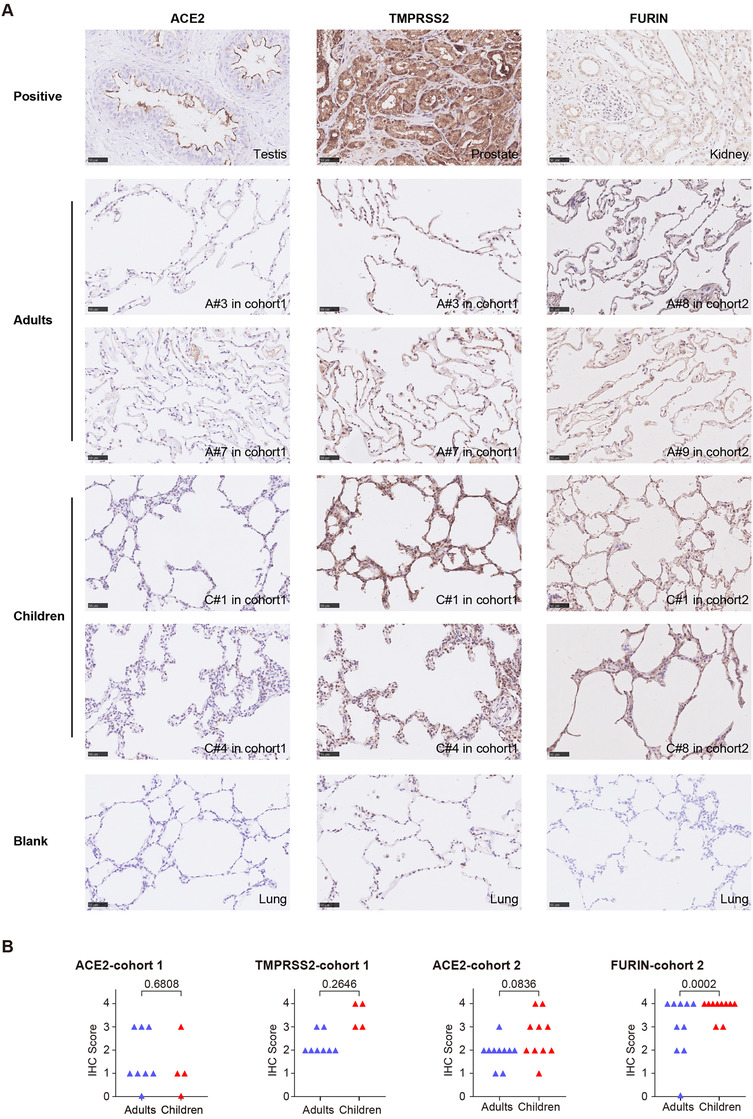
Expression of ACE2, TMPRSS2, and FURIN in the lung samples from children and adults determined by immunohistochemistry (IHC). Sections (4 μm) of formalin‐fixed, paraffin‐embedded (FFPE) lung tissues from adults and children were subjected to standard IHC protocols, in order to analyze the expression levels of ACE2 and TMPRSS2 (cohort 1) as well as ACE2 and FURIN (cohort 2). (A) The graphs are IHC representatives from both cohorts, with the patient number indicated. The tissues for positive controls of various antibodies are testis for ACE2, prostate for TMPRSS2, and kidney for FURIN. (B) Staining intensities for each antibody were evaluated in a semiquantitative, five‐tier manner (negative = 0, partial weak positive = 1, diffused weak positive = 2, partial strong positive = 3, and diffused strong positive = 4), independently by two pathologists who were blinded to the sample group. Mann‐Whitney test was used to compare the difference between children and adults for each protein, with an exact *p* value being indicated.

Although children of all ages are susceptible to SARS‐CoV‐2 infection, clinical data indicated better clinical outcomes in children compared to adults.^1^ However, the expression levels and the functional activity of ACE2 during child development remain largely unclear. Moreover, interpretations on available data from animal models remain controversial. The expression of *ACE2* reportedly decreases in the lungs during aging, according to a study performed in aged rats.^9^ It has also been shown that higher expression of *ACE2* was observed in the pulmonary alveolar epithelial barrier, cardiomyocytes, and vascular endothelial cells in aged non‐human primates.^10^

The present study showed comparable expression levels of ACE2, as well as other factors involved in SARS‐CoV‐2 cellular entry, in children and adults, suggesting that the expression level of genes associated with viral‐entry may not be the key reason explaining the milder symptoms observed in children. Instead, it is likely that other factors, such as unique features in children immunity, may play a more important role. Children have a distinct immune profile during infections because their immune system is still under development. It has been shown that, compared to adults, children have a decreased mononuclear and polymorphonuclear chemotaxis, and that such decrease remains significant until the age of 16.^5^ At the same time, frequent exposure to a plethora of other pathogens, such as respiratory syncytial virus, has the potential to repeatedly challenge innate immunity, including the age‐dependent maturation of interferon response.^6^ This likely leads to an enhanced innate immune function that is one of the features of trained immunity.^4^ Last but not least, this study provided a comparison of the basal levels of these genes in adults and children, and therefore cannot exclude the possibility that, upon viral infection, there could be different responsiveness of these genes in the two groups.

In summary, the present study described, for the first time, the features of children's lungs by scRNA‐seq. Both scRNA‐seq and IHC analyses showed a comparable (if not higher) level of expression of the key genes associated with the entry of SARS‐CoV‐2 in the cell, including ACE2, TMPRSS2, and FURIN. This suggests that rather than a lower virus invasion rate, there are other factors which are more likely to explain the milder symptoms of SARS‐CoV‐2 in infected children, which awaits and warrants further investigation.

## CONFLICT OF INTEREST

The authors declare that there is no conflict of interest that could be perceived as prejudicing the impartiality of the research reported.

## AUTHOR CONTRIBUTIONS

Yue Tao, Ruwen Yang, Chen Wen, Hao Zhang, Huiwen Chen, and Xi Mo designed the experiments and wrote the manuscript. Hao Chen and Guocheng Shi provided lung tissues from children for scRNA‐seq. Zhiguang Zhao and Xinyu Song provided the FFPE sections of adults’ lung tissues for IHC. Minzhi Yin provided the FFPE sections of children's lung tissues for IHC. Qiao He performed IHC. Jing Ma and Minzhi Yin evaluated the IHC score. Ruwen Yang, Jue Fan, and Nan Fang analyzed the data from scRNA‐seq. All authors participated in the data analysis and manuscript preparation.

## Supporting information



Supporting Information

Supporting Information

Supporting Information

Supporting Information

## References

[1] Swann OV , Holden KA , Turtle L , et al. Clinical characteristics of children and young people admitted to hospital with covid‐19 in United Kingdom: prospective multicentre observational cohort study. BMJ. 2020;370:m3249. 32960186 10.1136/bmj.m3249 PMC7488201

[2] Bi Q , Wu Y , Mei S , et al. Epidemiology and transmission of COVID‐19 in 391 cases and 1286 of their close contacts in Shenzhen, China: a retrospective cohort study. Lancet Infect Dis. 2020;20:911–919. 32353347 10.1016/S1473-3099(20)30287-5 PMC7185944

[3] Cristiani L , Mancino E , Matera L , et al. Will children reveal their secret? The coronavirus dilemma. Eur Respir J. 2020;55:2000749. 32241833 10.1183/13993003.00749-2020 PMC7113798

[4] Brodin P . Why is COVID‐19 so mild in children?. Acta Paediatr. 2020;109:1082–1083. 32212348 10.1111/apa.15271

[5] Klein RB , Fischer TJ , Gard SE , et al . Decreased mononuclear and polymorphonuclear chemotaxis in human newborns, infants, and young children. Pediatrics. 1977;60:467–472. 905012

[6] Simon AK , Hollander GA , McMichael A . Evolution of the immune system in humans from infancy to old age. Proc Biol Sci. 2015;282:20143085. 26702035 10.1098/rspb.2014.3085 PMC4707740

[7] Hoffmann M , Kleine‐Weber H , Schroeder S , et al. SARS‐CoV‐2 cell entry depends on ACE2 and TMPRSS2 and is blocked by a clinically proven protease inhibitor. Cell. 2020;181:271–280. 32142651 10.1016/j.cell.2020.02.052 PMC7102627

[8] Hamming I , Timens W , Bulthuis MLC , Lely AT , Navis GJ , van Goor H . Tissue distribution of ACE2 protein, the functional receptor for SARS coronavirus. A first step in understanding SARS pathogenesis. J Pathol. 2004;203:631–637. 15141377 10.1002/path.1570 PMC7167720

[9] Xie X , Chen J , Wang X , Zhang F , Liu Y . Age‐ and gender‐related difference of ACE2 expression in rat lung. Life Sci. 2006;78:2166–2171. 16303146 10.1016/j.lfs.2005.09.038 PMC7094566

[10] Ma S , et al. Single‐cell transcriptomic atlas of primate cardiopulmonary aging. Cell Res. 2020;10:1–18. 10.1038/s41422-020-00412-6 PMC7483052 32913304

