# Supplementary material for: Preliminary analyses of scRNA sequencing and immunohistochemistry of children's lung tissues indicate the expression of SARS‐CoV‐2 entry‐related genes may not be the key reason for the milder syndromes of COVID‐19 in children
Source: Clin Transl Med. 2021 May 21;11(5):e300. doi: 10.1002/ctm2.300 (PMC8140187; doi:10.1002/ctm2.300)
Supplement: Supplementary file 1 — Supporting Information [file CTM2-11-e300-s001.docx]

**SUPPLEMENTARY INFORMATION**

**METHODS**

**Human subjects and specimens**

Normal lung tissues from children and adults undergoing biopsies or surgeries for various reasons were collected for scRNA-seq and IHC analyses (Table S1). This study was approved by the Institutional Review Board and the Ethics Committee of Shanghai Children’s Medical Center (SCMCIRB-K2020034). Written informed consent was obtained from (the parents of) each patient.

**Tissue dissociation and single cell suspension preparation**

Fresh tissue samples were collected and immediately stored in the GEXSCOPETM Tissue Preservation Solution (Singleron Biotechnologies) at 4 °C. Prior to tissue dissociation, the specimens were washed three times with the Hanks Balanced Salt Solution (HBSS) and then minced into 1–2 mm pieces. The tissue pieces were digested in 2 ml of the GEXSCOPE Tissue Dissociation Solution (Singleron Biotechnologies) at 37 °C for 15 min in a 15 ml centrifuge tube with continuous agitation. After digestion, a 40-micron sterile strainer (Corning) was used to separate cells from cell debris and other impurities. The cell suspension was centrifuged at 1000 rpm at 4 °C for 5 min, and the cell pellets were resuspended in 1 ml PBS (HyClone). In order to remove red blood cells, 2 ml of the GEXSCOPE Red Blood Cell Lysis Buﬀer (Singleron Biotechnologies) was added to the cell suspension and incubated at 25 °C for 10 min. The mixture was then centrifuged at 1000 rpm for 5 min and the cell pellet was resuspended in PBS. The cells were counted with a TC20 automated cell counter (Bio-Rad) before further analyses.

**Single cell RNA sequencing (scRNA-seq) library preparation**

Single-cell suspension was adjusted to a concentration of 1×10^5^ cells/ml in PBS, and was then loaded onto a microfluidic chip. The scRNA-seq libraries were constructed according to the manufacturer’s instructions (Singleron GEXSCOPETM Single Cell RNA-seq Library Kit, Singleron Biotechnologies, www.singleronbio.com), and then sequenced on an Illumina HiSeq X Ten instrument with 150 bp paired end reads. We obtained an average sequencing depth per library around 61 thousand reads per cell.

**Primary analysis of** **scRNA-seq raw sequencing data**

Raw reads were processed to generate gene expression matrices by scopetools ([e](https://anaconda.org/singleronbio/scopetools)). Briefly, FastQC (V0.11.7), fastp (1), STAR aligner (2.5.3a) and featureCounts (1.6.2) were used for quality evaluation, trimming, alignment and transcript counting, respectively. We filtered out reads without poly(T) tails at the intended positions, and extracted the cell barcode and unique molecular identifier (UMI) for each read. Adapters and poly(A) tails were trimmed before aligning reads to the GRCh38 reference genome with the Ensembl 92 annotation. Reads with the same cell barcode, UMI and gene were grouped together to generate the number of UMIs per gene per cell. The cell number was then determined based on the inflection point of the number of UMI versus the sorted cell barcode curve. The scRNA-seq data have been deposited in the NCBI’s Gene Expression Omnibus database and are accessible through the GEO series accession number GSE155900.

**Integrated analysis of children lung data with public adult lung data**

The public data of 8 healthy adults were downloaded from the Gene Expression Omnibus database (GSE122960). We used Canonical Correlation Analysis (CCA) implemented in Seurat V3 (https://github.com/satijalab/seurat) to integrate the datasets from adults and children. First, we filtered out cells with less than 200 and more than 5000 genes identified, as well as those with less than 30000 UMIs. We also removed cells with a proportion of mitochondrial genes higher than 20%. 2000 highly variable genes were used for principal component analysis (PCA) and the first 20 PCs were selected for subsequent analysis. Resolution 1.25 was used for the clustering analysis to obtain 32 clusters, which were assigned to 15 major cell types based on their canonical marker genes.

**Immunohistochemistry (IHC)**

Sections (4 μm) of formalin-fixed, paraffin-embedded (FFPE) lung tissues were subjected to standard IHC protocols in order to analyze the expression levels of ACE2, TMPRSS2 and FURIN. After deparaffinization, antigen retrieval and blocking, the slides were stained using the Leica Bond automated staining system (Leica Biosystems), for 60 min at room temperature with the following primary antibodies: anti-ACE2 antibody (rabbit, ab108252 from Abcam, 1:100) and anti-TMPRSS2 antibody (rabbit, ab92323 from Abcam, 1:2000) for cohort 1; anti-ACE2 antibody (mouse, 66699-1-Ig, Proteintech, 1:1200) and anti-FURIN antibody (rabbit, 18413-1-AP, Proteintech, 1:1200) for cohort 2. After three washes, the slides were incubated with either HRP-conjugated goat anti-rabbit antibody (cohort 1) or biotin-conjugated anti-mouse/rabbit secondary antibody (cohort 2) for 15-30 min at room temperature. After DAB staining, the slides were counterstained with hematoxylin, dehydrated and mounted. Staining intensities for each antibody were evaluated in a semiquantitative, five-tier manner (negative = 0, partial weak positive = 1, diffused weak positive = 2, partial strong positive = 3, diffused strong positive = 4), independently by two pathologists that were blinded to the sample group. The inconsistent scores were determined after consulting the two pathologists.

**Statistical analysis and data visualization**

UMAP (Uniform manifold approximation and projection) visualization of an integrated scRNA-seq dataset includes data from GSE122960 and the sequencing results from ten children lung specimens. 15 clustered and annotated cell types are color-coded, while cells from adults and children are represented with blue and red dots, respectively. We applied a *t*-test to compare the expression levels of *ACE2*, *TMPRSS2* and *FURIN* between children and adults determined by the scRNA-seq analysis. The comparison was performed based on library size normalized counts. The normalization was done using the NormalizeData function in Seurat V3. The R package ggpubr was used to perform *t*-tests and produce boxplots for visualization. The dot plot was generated using the Seurat V3 DotPlot function. Dot color represents the Z-score normalized average gene expression within each particular cell type. Dot size represents the percentage of cells expressing the respective genes within each cell type. Statistical analyses of IHC scores were performed in GraphPad Prism 8 using the Mann-Whitney test with a computed exact *p* value. Figures were generated in GraphPad Prism 8. A *p* value lower than 0.05 was considered as statistically significant.

**SUPPLEMENTARY TABLES**

Table S1. Basic characteristics of children and adults used for single cell RNA-seq of lung tissues.

Table S2. Basic characteristics of children and adults used for immunohistochemistry of lung tissues.

Table S3. Expression of *ACE2*, *TMPRSS2* and *FURIN* across all 15 cell types in lung samples.
